# High Seroprevalence of Bluetongue Virus Serotype 3 in Belgian Cattle and Sheep After the 2024 Epidemic

**DOI:** 10.3390/v18030396

**Published:** 2026-03-22

**Authors:** Mickaël Cargnel, Xavier Simons, Ilse De Leeuw, Nick De Regge, Jean-Baptiste Hanon

**Affiliations:** 1 Sciensano, Infectious Diseases in Animals, Coordination of Veterinary Activities and Veterinary Epidemiology, 1050 Brussels, Belgium; xavier.simons@sciensano.be (X.S.); jean-baptiste.hanon@sciensano.be (J.-B.H.); 2 Sciensano, Infectious Diseases in Animals, Exotic and Vector-Borne Diseases, 1050 Brussels, Belgium; ilse.deleeuw@sciensano.be (I.D.L.); nick.deregge@sciensano.be (N.D.R.)

**Keywords:** bluetongue virus, BTV-3, national surveillance programme, within-herd seroprevalence

## Abstract

To monitor the epidemiological situation of bluetongue virus (BTV) in Belgium, a national surveillance programme was conducted during the 2024–2025 winter season. The objective was to estimate the apparent seroprevalence of BTV-3 following the 2023–2024 epidemic and to prove the absence of active circulation of other BTV serotypes in mixed herds (cattle and sheep). A total of 2551 cattle and 1458 sheep were sampled across Belgium. Serological analyses were performed using ELISA, and molecular detection of BTV-3, BTV-8, and BTV-12 was conducted by RT-qPCR. The majority of cattle and sheep herds showed evidence of exposure to BTV-3, with a very high herd-level apparent seroprevalence (100%; 95% CI: 96.2–100% in cattle and 98.9%; 95% CI: 93.8–99.8% in sheep). Apparent within-herd seroprevalence was also high in cattle (94.6%; 95% CI: 91.8–96.5%) and sheep (85.5%; 95% CI: 80.4–89.5%). No evidence of active circulation of BTV-8 or BTV-12 was detected. A moderate significant positive correlation between Ct values and sampling date was observed both for bovine and ovine samples, consistent with a progressive decline in detectable BTV RNA during winter in the absence of vector activity.

## 1. Introduction

Bluetongue (BT), also known as ovine catarrhal fever, is a non-contagious arthropod-borne disease affecting domestic and wild ruminants. It is caused by bluetongue virus (BTV), a segmented double-stranded RNA virus belonging to the genus *Orbivirus* (SedoReoviridae) [1,2,3]. To date, at least 36 BTV serotypes have been identified globally [4], with serotypes 1–24 listed as officially notifiable under the European Animal Health Law (Regulation EU 2016/429) [5] and by the World Organisation of Animal Health (WOAH) (WOAH terrestrial code, chapter 1.3). Transmission primarily occurs via bites from *Culicoides* spp. midges. Clinical manifestations may occur in acutely infected domestic ruminants but are most severe in sheep, characterized by oedema, tongue swelling, inflammation of coronary bands, hyperaemia, apathy, hypersalivation, nasal discharge, mucosal erosions and ulcerations, lameness, and high mortality [6,7,8]. Infection in cattle and goats is often subclinical or associated with milder clinical signs, although goats usually experience milder clinical symptoms [7].

BT causes substantial economic losses, both direct and indirect. Direct losses arise from high morbidity and mortality, abortions, stillbirths, congenital abnormalities, low birth weight, reduced milk yield, lowered fertility, early culling, and meat and fleece losses. Indirect losses stem from trade restrictions on animals, germplasm, and animal products, as well as costs associated with vaccination, diagnosis, vector control, and treatment of affected animals [9].

In Europe, the circulation of bluetongue virus (BTV) was historically mostly restricted to the Mediterranean Basin. However, BTV serotype 8 (BTV-8) emerged in 2006 in the Netherlands and caused a major epidemic between 2006 and 2010, rapidly spreading across Belgium, Germany, France, and Luxembourg [9,10,11,12]. Between 2010 and 2023, Northern Europe experienced several BTV outbreaks, including the re-emergence of BTV-8 and incursions of other serotypes [11]. Notably, genetically distinct strains within the same serotype have been identified in Europe, reflecting the evolutionary dynamics of BTV.

In Belgium, control measures including mass vaccination campaigns and a strict surveillance programme combined with the absence of clinical cases and virus circulation since 2009 enabled the country to regain its official BTV-free status in 2012, in accordance with European Commission Regulation (EC) No 2020/689 [13]. This free status was maintained until 2018 and reinstated again from 5 June 2023.

In 2023, an outbreak of BTV-3 started in the Netherlands [14] and a limited number of BTV-3 outbreaks were detected in Belgium during the autumn of the same year, resulting in the loss of its BTV-free status since 10 October 2023. In contrast to this limited initial incursion, a widespread BTV-3 epidemic affected Belgium from July 2024 onwards [11] with 2534 outbreaks officially reported in cattle herds and 1046 in sheep flocks [15]. This epidemic caused unprecedented mortality among sheep, far exceeding previous BTV outbreak impacts in Europe [16]. In addition to BTV-3, BTV-12 has also emerged as a concern in Europe [17]. Although previously considered sporadic and geographically limited, BTV-12 infections were reported in several Northern European countries in 2023–2024, including countries with which Belgium has substantial cattle trade exchanges, thereby increasing the potential epidemiological relevance for Belgium. The clinical manifestations in affected ruminants appear generally milder than those observed during the BTV-3 epidemic, but infections have been associated with reproductive losses in cattle and transient morbidity in sheep [17]. When BTV-3 emerged in the Netherlands and then in Belgium in 2023, no commercial vaccines were authorized and available. In the first half of 2024, three new commercial vaccines were authorized to be used by private veterinarians to vaccinate cattle and sheep. This vaccination was however limited since it was performed on a voluntary basis and at the farmers’ cost. In addition, the vaccine was only available in May, while the majority of animals were already on pasture, which complicated administration of the two-dose primary vaccination. Following the epidemic of BTV-3 in Belgium in 2024, and the threat of re-emergence of BTV-8 as a result of the northwards spread of this serotype in neighbouring France, vaccination against BTV-3 and BTV-8 in sheep and cattle was made mandatory in Belgium in 2025. This vaccination targeted all animals born before 1 January 2025 (except fattening calves) to protect herds and limit virus spread in naïve animals. No vaccine is currently available for BTV-12.

This article uses data generated by the Belgian national annual surveillance campaign conducted during the 2024–2025 vector-free season, which was specifically designed to address the epidemiological situation following the recent BTV-3 epidemic and therefore primarily targeted BTV-3 circulation. Specifically, the study aimed to (i) estimate the herd and within-herd apparent seroprevalence of BTV-3 in cattle and sheep populations, and (ii) assess the absence of active circulation of BTV-8 and BTV-12 during the winter 2024–2025 period. By doing so, it provides a comprehensive overview of BTV circulation in Belgium.

## 2. Materials and Methods

### 2.1. Sample Size

The sampling strategy and sample size were designed to take into account the double objective of the survey: (i) estimate the herd and within-herd seroprevalence of BTV-3 and (ii) confirm the absence of circulation of BTV-8 and BTV-12 (i.e., maintain the BTV-free status of Belgium for non BTV-3 serotypes, especially BTV-8 and 12).

#### 2.1.1. Sample Size to Estimate the Apparent Herd Seroprevalence of BTV-3

The sample size required us to estimate the apparent herd-level seroprevalence (i.e., the proportion of herds infected) was calculated using the standard formula for estimating a proportion [18] (Equation (1)).

(1) $$n_{\mathrm{herds}}=\frac{\left(Z_{1-\frac{\alpha }{2}}^{2}\;p\;\left(1-p\right)\right)}{d^{2}}$$

where

*Z* is fixed at 1.96 and corresponds to the standard normal deviate for a 95% confidence level.

*p* is the expected herd-level prevalence set at 75%. At the time of calculation, the only available data came from the Netherlands, reporting a herd-level prevalence of 64% [8]. Considering historical patterns of BTV outbreaks in Belgium, such as the BTV-8 epidemic in 2006, which were characterized by extremely high herd-level prevalence, we considered 75% to be a reasonable assumption for the expected herd-level prevalence in our study.

*d* is the desired absolute precision fixed at 5%.

Under a simple random sampling design, the required sample size was estimated at 289 animals from different herds to assess the apparent herd-level seroprevalence of BTV-3. For practical reasons, including the reduction in farm visits and associated costs, a two-stage cluster sampling design was implemented, with herds defined as primary sampling units and animals as secondary sampling units. Specifically, mixed farms (i.e., holdings with both cattle and sheep) were selected, and within each herd, 30 cattle and 20 sheep were sampled. However, when multiple animals are selected from the same farm, the total sample size must be adjusted to account for the intra-cluster correlation [19] (Equation (2)).

(2) $$n_{\mathrm{herds}}^{\prime }=(n_{\mathrm{herds}}\cdot (1+\rho (m-1)))/m$$

where

n′ is the adjusted sample size.

n is the original sample size (i.e., 289 herds).

ρ is the intra-cluster correlation coefficient (ICC), as animals within the same herd are not independent. We arbitrary fixed the ICC at 31%, which lies between two reported values for BTV: 21% [20] and 41% [21].

m is the number of animals sampled per farm (fixed at 20 to increase sample size).

Based on this design, sampling at least 20 animals in 100 herds is required to achieve the desired precision. This result was confirmed using an online two-stage sampling sample size calculator, accounting for clustering developed by the University of Melbourne (Australia), available at https://shiny.vet.unimelb.edu.au/epi/samplesize/ (accessed 1 February 2026).

#### 2.1.2. Sample Size to Estimate the Apparent Within-Herd Seroprevalence of BTV-3

The sample size per herd required us to estimate the apparent within-herd seroprevalence (i.e., the proportion of infected animals in infected herds) was calculated at the animal level using the adjusted formula for estimating a proportion [18] (Equation (3)):

(3) $$n_{\mathrm{within}\;\mathrm{herd}}=\frac{\left(Z_{1-\frac{\alpha }{2}}^{2}\;p_{w}\;\left(1-p_{w}\right)\right)\times N}{d_{w}^{2}\times \left(N-1\right)+Z_{1-\frac{\alpha }{2}}^{2}\;p_{w}\;\left(1-p_{w}\right)}$$

where

*Z* is fixed at 1.96 and corresponds to the standard normal deviate for a 95% confidence level.

$p_{w}$ is the expected apparent within-herd seroprevalence. In the absence of *prior* data on within-herd seroprevalence of BTV-3 in Belgium, the expected apparent within-herd prevalence was set at 20%, based on observations from the Netherlands [22].

$d_{w}$ is the desired absolute precision fixed at 10% for cattle and 15% for sheep.

*N* represents the finite population size within herds. In Belgium, the average herd size was estimated at 53 cattle per herd [23]. For sheep, 121,377 animals were reported across in 2744 professional herds in 2023, corresponding to an average of 44 sheep per herd [24].

This resulted to a theoretical requirement of 29 cattle and 26 sheep per herd, assuming simple random sampling. For budgetary reasons, the number of cattle was set at 30 per herd, and the number of sheep at 20 per herd. This led to a slightly lower desired precision of 13.5% in sheep instead of the originally planned 10%.

#### 2.1.3. Sample Size to Assess the Absence of Active Circulation of BTV-8 and BTV-12

Although the surveillance design and sample size were primarily determined to estimate BTV-3 within-herd and herd-level seroprevalence, the collected samples were secondarily used to demonstrate freedom from BTV-8 and BTV-12, based on a structured surveillance framework derived from the principles of detection commonly used in freedom-from-disease designs. Although Belgium was not officially bluetongue-free at the time of the study, this approach was adopted to provide a harmonized and conservative framework to detect widespread virus circulation across the territory. The design prevalence was chosen as a pragmatic threshold to identify substantial viral circulation rather than to demonstrate official freedom from infection. According to Commission Delegated Regulation (EU) 2020/689 (Annex V, Part II, Chapter 4, Section 2: Maintenance of disease-free status), specific rules apply for a Member State to maintain its “bluetongue-free” status, in this case for serotypes BTV-8 and BTV-12, which were the serotypes present in the neighbouring countries at the time of the study (France and The Netherlands, respectively). It was based on spatial representativeness across Belgium using a 45 km × 45 km grid system and must be able to detect infection with at least a 95% confidence level at a target prevalence of 20% to maintain disease-free status.

Sample size was calculated using Epitools, an online platform offering statistical calculators for epidemiological analysis, available at: http://epitools.ausvet.com.au [25]. We used the “Sample size to achieve specified population level (or herd, flock, cluster, etc.) sensitivity” tool (https://epitools.ausvet.com.au/freedomss) (accessed 1 February 2026) and the following parameters were applied: a design prevalence of 20%, based on observations from the Netherlands [22], a required population sensitivity of 95%, and an unknown population size. Test sensitivity was set at 98% to account for potential variability in field conditions, consistent with a conservative assumption relative to the reported diagnostic sensitivity of the enzyme-linked immunosorbent assay used (Pan-ELISA; ID Screen^®^ Bluetongue Competition assay, ID.VET, Montpellier, France; sensitivity 100%, 95% CI: 99.49–100%) [26]. All positive ELISA were confirmed by RT-qPCR (see Section 2.3.2). This resulted in a required sample size of 14 cattle and 14 sheep per 45 km × 45 km grid ($n_{animals})$. As previously mentioned, a cluster sampling design was applied, targeting mixed farms, with 30 cattle and 20 sheep sampled per herd. The total sample size ($n_{animals}^{\prime })$ was adjusted to account for intra-cluster correlation using the following formula [19] (Equation (4)).

(4) $$n_{\mathrm{animals}}^{\prime }=n_{\mathrm{animals}}\;(1+\rho (m-1))$$

where

$n_{\mathrm{animals}}^{\prime }$ is the adjusted sample size per 45 km × 45 km grid;

$n_{\mathrm{animals}}$ is the original sample size per 45 km × 45 km grid;

ρ is the intra-cluster correlation coefficient (ICC), fixed the ICC at 31% (see Equation (1));

m is the number of animals sampled per farm (30 cattle and 20 sheep).

Based on this adjustment, five farms per grid were required, corresponding to a total of 140 cattle and 96 sheep sampled per grid. However, the available budget allowed the inclusion of seven farms (210 cattle and 140 sheep) per 45 km × 45 km grid, which was therefore implemented.

As all the analysis was to be conducted at the provincial level, we estimated how many 45 km × 45 km grids would be needed to represent each Belgian province. This was done using a simple proportional rule based on surface area. For example, the province of Liège covers 3857 km^2^, which corresponds to approximately 2 grids of 45 km × 45 km (i.e., 2025 km^2^ per grid).

The number of herds to be sampled was determined, in each province (Table 1), as follows (Equation (5)):

(5) $$\mathrm{Number}\;\mathrm{of}\;\mathrm{herds}\;\mathrm{to}\;\mathrm{be}\;\mathrm{sampled}=\frac{\mathrm{number}\;\mathrm{of}\;\mathrm{grids}\;\mathrm{in}\;\mathrm{the}\;\mathrm{province}}{15}\times 100\mathrm{herds}$$

where

15 is the total number of 45 km × 45 km grids covering Belgium’s surface area and 100 is the total number of herds to be sampled in Belgium (Table 1).

### 2.2. Herds Selection

On 18 October 2024, data related to all active mixed herds (i.e., herds keeping both cattle and sheep) were extracted from SANITEL, the Belgian national animal identification and registration database. Mixed herds were selected to allow the simultaneous estimation of prevalence in both species, while limiting the total number of herds to be visited. For each herd, the following information was retrieved: the herd identification number, the geographic location, vaccination dates, and the vaccine brand. Individual-level data on animal movements and date of birth were collected for cattle and, for sheep, the number of sheep recorded in the herd at the most recent inventory (15 December 2023). For sheep, although individual identification is mandatory, movements are not recorded at the animal level but are reported at the group level.

Eligible herds were mixed herds containing cattle aged 12–60 months born in the herd and with sheep aged ≥6 months. The lower age limit excluded animals potentially protected by maternal antibodies (reducing false-positive risk) [27,28], while the upper limit reduced the risk of detecting residual antibodies from exposure prior to the officially recognized period of freedom or due to vaccination more than 5 years before the sampling. No specific age limit was set for sheep, as animals older than 60 months are uncommon in professional sheep farms, given that older ewes are typically culled due to declining productivity. The upper age limit of 60 months was selected to minimize the likelihood of detecting residual antibodies from historical exposure to BTV circulation prior to the recent re-emergence of BTV-3 in 2023, and to ensure that seropositivity primarily reflected recent infection or vaccination associated with the 2024 epidemic. Since the objective was to detect seropositive animals to estimate seroprevalence of BTV-3 (due to infection or to BTV-3-vaccination), herds that reported BTV vaccination since 1 January 2019 against a serotype other than BTV-3 were excluded.

Herds were selected randomly in each province according to the distribution described above, using SAS software (version 9.4, SAS Institute Inc., Cary, NC, USA) to achieve the required number (*n* = 100).

### 2.3. Sampling and Laboratory Testing

The surveillance programme for BTV was performed during the winter season 2024–2025, known as “winter screening” (WS). Animals that fulfilled the eligibility criteria (i.e., age, herd of birth for cattle, vaccination) were chosen in the selected herds by the regional veterinary laboratories (Association Régionale de Santé et d’Identification Animales *(ARSIA)* and the Dierengezondheidszorg Vlaanderen (DGZ). Individual blood samples were collected by the farm veterinarians as clotted blood for serum preparation and as EDTA-anticoagulated blood. Samples for this screening were collected during winter (from December 2024 to April 2025), when the absence of midge vector activity (*Culicoides*) allows detection of infections acquired during the previous transmission season.

#### 2.3.1. Enzyme-Linked Immunosorbent Assay+

Samples were conditioned to serum at the regional laboratories of ARSIA and DGZ. The serum samples were analyzed using ELISA (ID Screen^®^ Bluetongue Competition assay, ID.VET, Montpellier, France) according to the manufacturer’s instructions. This competitive ELISA was validated [29] and showed a measurement sensitivity of 100% (95% confidence interval (CI): 99.49–100%, *n* = 754 cattle, sheep, goats) and a measurement specificity of 100% (95% CI: 99.84–100%, *n* = 2461 cattle, sheep, goats) [26]. Results were expressed as percentage negativity (PN% = [optical density sample/optical density negative kit control] × 100). Samples with PN < 40%, and ≥40% were considered positive and negative, respectively. Non-interpretable results due to poor sample quality were excluded from the final dataset.

#### 2.3.2. Real-Time Quantitative Polymerase Chain Reaction

ELISA-positive animals were tested by RT-qPCR on EDTA blood samples at the Belgian National Reference Laboratory (Sciensano) to confirm freedom from disease. EDTA blood samples were pooled by herd and further by animal species (cattle or sheep) up to a maximum of 10 individual samples.

RNA was extracted from these pooled samples using the Indimag 48 extraction robot with the Indimag Pathogen Kit (INDICAL BIOSCIENCE GmbH, Leipzig, Germany), according to the manufacturer’s instructions.

Extracted RNA was analyzed using a duplex pan-BTV real-time RT-qPCR assay targeting segment 10 encoding the NS3 protein, as described by Hofmann et al. (2008) [30], combined with an endogenous control reaction targeting GAPDH [11]. Amplification was performed using the AgPath-ID One-Step RT-PCR Kit (Thermo Fisher, Merelbeke, Belgium) under the following cycling conditions: 45 °C for 10 min, 95 °C for 10 min, followed by 45 cycles of 95 °C for 15 s and 56 °C for 45 s. Samples with Ct values ≤38 and showing typical exponential amplification curves were considered positive, whereas samples with Ct values >38 and <45 were classified as doubtful. Samples without amplification or with Ct values ≥45 were considered negative. A Ct value <35 for the endogenous control (GAPDH) was required to validate a sample.

Serotype-specific real-time RT-qPCR assays targeting segment 2 encoding the outer capsid protein VP2 were performed for BTV-3, BTV-8, and BTV-12 using published protocols [31,32,33]. These serotype-specific RT-qPCR assays were performed on all samples tested by pan-BTV RT-qPCR, including those with negative, doubtful, and positive pan-BTV results. These assays were run using the same reaction mix as the pan-BTV RT-qPCR, but with an annealing temperature of 60 °C.

### 2.4. Statistical Analysis

All analyses were performed using SAS software (version 9.4, SAS Institute Inc., Cary, NC, USA).

#### 2.4.1. Data Cleaning and Descriptive Analysis

For each sample result, animal eligibility was verified using SANITEL data. Information on date of birth and movements was used to confirm compliance with age criteria and to ensure cattle were born in the herd where they were sampled. This information is not available for sheep. Vaccination status against BTV-3 for each animal was assessed from records in SANITEL, where BTV vaccination is recorded by the veterinarian at the herd level for both cattle and sheep. In cattle, animals were considered potentially vaccinated if they were born before the herd vaccination date and were present in the herd during vaccination, and if at least six days had passed between vaccination and sampling, allowing sufficient time for antibody titres to rise. In sheep, herds were considered potentially fully vaccinated if sampling occurred at least six days after the herd vaccination date. As we target specifically BTV-3, animals or herds without a recorded BTV-3 vaccination were classified as non-vaccinated.

The descriptive analysis summarized the number of animals sampled per herd and per province, their age distribution, and their distribution across regions. Animals not meeting the predefined age requirements or with inconsistent identification were excluded. The proportion of cattle born in the herd where they were sampled was also calculated to further evaluate compliance with the eligibility criteria.

#### 2.4.2. Apparent Seroprevalence Calculation and Comparison Between Provinces

Herd-level apparent prevalence at the national level, together with 95% CI, was calculated using Epitools (module “Confidence limits for a proportion”; https://epitools.ausvet.com.au/ciproportion) (accessed 1 February 2026), with confidence intervals calculated using the Wilson method, which provides good coverage properties for population-level proportions.

Within-herd apparent seroprevalence was estimated using PROC FREQ in SAS, with exact 95% binomial confidence intervals computed using the Clopper–Pearson method, a conservative and distribution-free approach well suited to small sample sizes and within-herd estimate.

To estimate this seroprevalence and to account for the clustering of animals within herds, we used a Generalized Estimating Equation (GEE). GEE is well-suited for correlated data arising from cluster sampling designs and provides robust standard error estimates even under misspecification of the correlation structure. A binomial distribution with a logit link function was specified, given the binary nature of the ELISA test results (positive or negative). An exchangeable correlation structure was assumed to model the intra-herd correlation among animals sampled within the same herd.

Sampling weights were incorporated into the analysis to adjust for the unequal probabilities of herd and animal selection inherent in the stratified cluster sampling design. Specifically, the selection probability for each animal was calculated as follows (Equation (6)):

(6) $$\mathrm{Selection}\;\mathrm{probability}=\frac{n}{N}\times \frac{m}{M}$$

where

n = number of herds sampled in a given stratum (e.g., country, region, or province);

N = total number of herds in the same stratum;

m = number of animals sampled in the stratum;

M = total number of animals present in the stratum (or estimated herd capacity for sheep).

The final weight assigned to each animal was the inverse of its selection probability. Weighted analyses provide prevalence estimates that are more representative of the target population, though they may lead to wider confidence intervals due to increased uncertainty associated with the sampling design.

To investigate geographic variation in within-herd apparent seroprevalence, region, province, and country (Belgium) were included as explanatory variables in the GEE model. This within-herd apparent seroprevalence calculation at the national level was carried out with and without the potentially vaccinated animals.

Finally, within-herd apparent seroprevalence was compared between provinces using GEE. Pairwise comparisons between provinces were performed using least-squares means with Tukey adjustment to control for Type I error inflation associated with multiple testing.

#### 2.4.3. Correlation Between RT-qPCR and Sampling Date

For each sample tested, RT-qPCR results were reported as cycle threshold (Ct) values. Lower Ct values indicate higher amounts of viral RNA, whereas higher Ct values correspond to lower viral RNA levels. The temporal relationship between quantitative RT-qPCR results and sampling date was first explored graphically by plotting individual Ct values against time. This visual inspection was used to assess overall dispersion and trends. Only samples with positive or doubtful RT-qPCR results (Ct < 45), as defined in the diagnostic criteria, were included in this analysis.

The association between Ct values obtained from the pan-BTV RT-qPCR and the BTV-3, BTV-8, and BTV-12 RT-qPCR assays and sampling date was quantified using correlation analyses. For each species, Pearson’s correlation coefficient was used to assess the presence of a linear association between Ct values and sampling date, whereas Spearman’s rank correlation coefficient was used to evaluate a monotonic association without assuming normality or linearity. Correlation coefficients and corresponding *p*-values were reported. Pearson’s correlation coefficient measures the strength and direction of a linear relationship between two variables, while Spearman’s rank correlation assesses a monotonic relationship that does not require linearity or normal distribution of the data. The associated *p*-values test the null hypothesis that there is no association between Ct values and sampling date. The use of both approaches (Pearson and Spearman) allowed us to assess the robustness of the observed temporal association to different statistical assumptions. Statistical significance was assessed at the 5% level. Analyses were performed for all data and by species.

## 3. Results

### 3.1. Cattle

#### 3.1.1. Descriptive Analysis

Out of 449 eligible mixed herds, 98 were sampled (out of the 100 planned) from December 2024 to March 2025 and were distributed across all Belgian provinces.

A total of 2639 cattle were sampled but, after data quality control, 87 cattle individuals were excluded: 79 because their age did not meet the inclusion criteria (78 older than 60 months and 1 younger than 12 months) and 8 due to incorrect or incomplete identification. The final dataset therefore consisted of 2551 cattle retained for analysis, while the number of herds remained unchanged at 98.

The distribution of sampled herds and animals across provinces is presented in Table 2. Sampling was geographically balanced, with both Flemish and Walloon provinces represented. However, fewer herds than expected were sampled in some Walloon provinces: Luxembourg and Namur (12 instead of 13). In the other provinces, the target number of herds was successfully sampled. At the animal level, the number of cattle included per province varied between 79 (Walloon Brabant) and 346 (Luxembourg).

**Figure 1:**
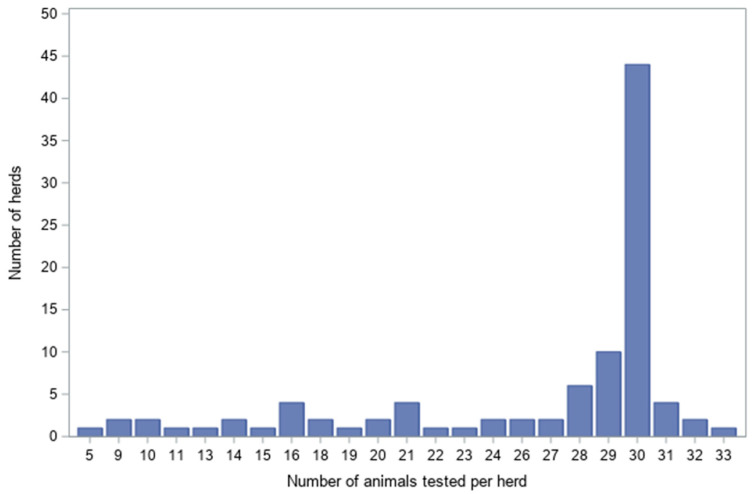
Distribution of herds by number of cattle sampled.

Within herds, sample size varied considerably. At least 30 animals were sampled in 51 herds (52.0%), whereas the smallest herd contributed only 5 animals. The maximum number of sampled animals per herd was 33 (Figure 1).

**Figure 2:**
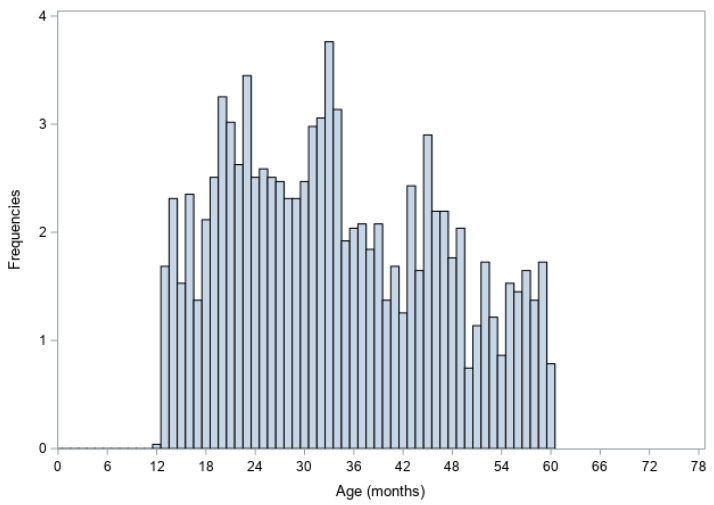
Age distribution of sampled cattle.

The mean age of sampled cattle was 34.0 months (standard deviation: 12.8 months). The age distribution was slightly right-skewed, reflecting a modest overrepresentation of younger animals (Figure 2). Assuming a symmetric distribution across the predefined inclusion range (12–60 months), the expected mean age would have been approximately 36 months, confirming that the observed dataset contained proportionally more younger cattle.

Serological results indicated an age-related increase in the proportion of ELISA-positive animals. The lowest seropositivity proportion was observed in the youngest age class (12–18 months, 76.0%), while older age classes exhibited progressively higher proportions: 93.7% in the 18–24 month class, 93.5% in the 26–36 month class, 96.0% in the 36–48 month class, and up to 97% in the 48–60 month class.

Regarding animal origin, the large majority of cattle (2417/2551; 94.7%) were born in the herd where they were sampled, limiting the potential impact of animal movements on exposure status and vaccination.

#### 3.1.2. Apparent Herd Seroprevalence

All sampled herds (98/98) tested ELISA-positive for BTV, with at least one positive animal per herd. Based on our sample, we can estimate that the BTV prevalence in the bovine herds in Belgium during that period is 100% with the corresponding 95% CI ranging from 96.2% to 100%.

#### 3.1.3. Apparent Within-Herd Seroprevalence Analysis

Overall, within-herd seroprevalence was very high at the national level (94.6%; 95% CI: 91.8–96.5%) (Table 3). At the regional level, Flanders had a slightly higher prevalence (95.5%; 95% CI 91.8–97.6%) than Wallonia (93.8%; 95% CI 89.1–96.5%), but the difference was not statistically significant as confidence intervals overlap. At the provincial level, prevalence varied: in Flanders, estimates ranged from 91.8% in East Flanders to 99.5% in Flemish Brabant, while in Wallonia, they ranged from 89.6% in Hainaut to 99.4% in Walloon Brabant. Other provinces also showed high within-herd prevalence, with some lower estimates associated with wider confidence intervals. However, there was no statistically significant difference in seroprevalence between provinces as shown by the pairwise comparison using the Tukey–Kramer adjustment (Table S1).

Twelve herds (12/98; 12.2%) contained animals potentially vaccinated against BTV-3. In these herds, 303 animals were identified as potentially vaccinated, of which 255 (83.2%) were tested seropositive. Among the vaccinated animals, 205 received Bultavo-3, 68 received Bluevac-3, and 30 received Syvazul BTV-3.

When examining the vaccination dates, eight herds were vaccinated with Bultavo-3. Among these, 119 animals received one dose, of which 89 (74.8%) were seropositive; 46 animals received two doses, of which 39 (84.8%) were seropositive; 16 animals received three doses, all of whom were seropositive; and 24 animals received four doses, all of whom were seropositive. Among the 68 animals vaccinated with Bluevac-3 in three herds (single dose), 61 (89.7%) were seropositive. For Syvazul BTV-3, among 30 animals vaccinated in one herd (single dose), 27 (90.0%) were seropositive.

When calculating national within-herd prevalence separately for unvaccinated (*n* = 86) and potentially vaccinated herds (*n* = 12), seroprevalence was high in both groups: 95.0% (95% CI: 92.1–96.9%) in unvaccinated herds and 89.0% (95% CI: 78.2–94.8%) in vaccinated herds; although within-herd prevalence was slightly higher in unvaccinated herds the difference was not statistically significant as confidence intervals overlap.

No statistically significant differences in within-herd prevalence were observed between provinces, based on pairwise comparisons with *p*-values adjusted using the Tukey method for multiple comparisons (Table S1). Adjusted *p*-values ranged from 0.09 to 1.00, indicating a high degree of homogeneity in within-herd prevalence across all provinces. The three lowest adjusted *p*-values were observed for comparisons involving Hainaut versus Flemish Brabant (adjusted *p*-value = 0.09), East Flanders versus Flemish Brabant (adjusted *p*-value = 0.15), and Liège versus Flemish Brabant (adjusted *p*-value = 0.15), but these did not reach the threshold for significance (0.05).

### 3.2. Sheep

#### 3.2.1. Descriptive Analysis

**Figure 3:**
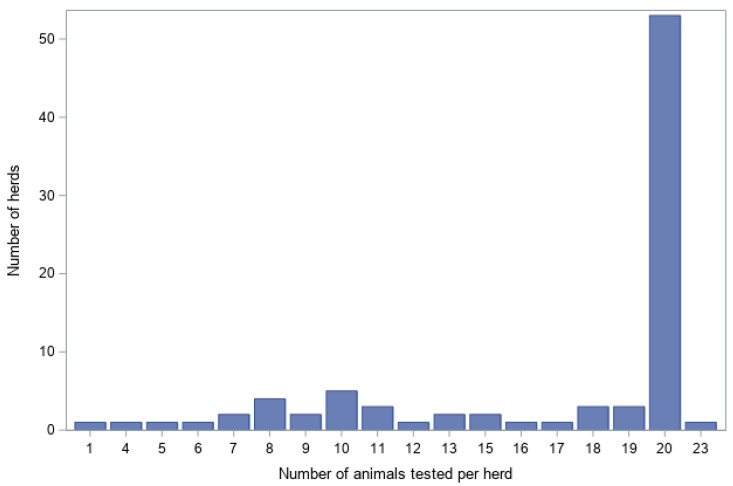
Distribution of herds by number of sheep sampled.

A total of 1458 sheep in 87 herds were sampled between December 2024 and April 2025 (Table 4). Fewer herds than expected were sampled in some provinces: Flemish Brabant (6 instead of 7), West Flanders (9 instead of 10), Hainaut (12 instead of 13), Liège (10 instead of 13), Luxembourg (10 instead of 13), and Namur (9 instead of 13). Sample sizes per herd met or exceeded the 20-animal target in 62.1% (54/87 herds) of herds (Figure 3).

#### 3.2.2. Apparent Herd Seroprevalence

Almost all herds tested positive for BTV-3 antibodies, with 98.9% (86/87) testing seropositive (95% CI: 93.8–99.8%). Only one herd, comprising 20 animals, was entirely seronegative.

#### 3.2.3. Apparent Within-Herd Seroprevalence Analysis

At the national level, the estimated within-herd apparent prevalence was 85.5% (95% CI: 80.4–89.5%). At the regional level, the prevalence was 84.0% (95% CI: 75.6–89.9%) in Flanders and 87.3% (95% CI: 81.5–91.5%) in Wallonia. At the provincial level, estimates ranged from 76.6% (95% CI: 59.6–87.9%) in Hainaut to 95.3% (95% CI: 92.0–97.2%) in Liège (Table 5).

The within-herd prevalence was estimated at 84.9% (95% CI: 78.9–89.4%) in unvaccinated herds (*n* = 16) and 88.2% (95% CI: 77.5–94.2%) in vaccinated herds (*n* = 71). Difference between both vaccinated and unvaccinated herds was not statistically significant, as the confidence intervals overlap.

Pairwise comparisons of within-herd prevalence between provinces revealed very few statistically significant differences after Tukey adjustment for multiple comparisons (Table S2). Most adjusted *p*-values were close to 1.00, indicating no meaningful difference in within-herd prevalence between the majority of provinces. However, a few exceptions were observed: a statistically significant difference was found between West Flanders and Liège (adjusted *p*-value = 0.02) and between West Flanders and Hainaut (adjusted *p*-value = 0.01). Marginally non-significant differences were observed between Liège and East Flanders (adjusted *p* = 0.08), as well as between Liège and Luxembourg (adjusted *p* = 0.09).

#### 3.2.4. Absence of Active Circulation of BTV-8 and BTV-12

Any ELISA-positive results were subsequently subjected to confirmatory PCR testing. No PCR-positive animals were detected in either cattle or sheep. Given that the sampling strategy was designed to achieve a population-level sensitivity of 95% at a design herd-level prevalence of 20%, the absence of confirmed positive results indicates, with at least 95% confidence, that the cattle and sheep population were free from infection at or above this prevalence threshold.

### 3.3. Correlation Between RT-qPCR and Time

As all RT-qPCR results for BTV-8 and BTV-12 were negative, correlation analyses were not performed for these serotypes.

**Figure 4:**
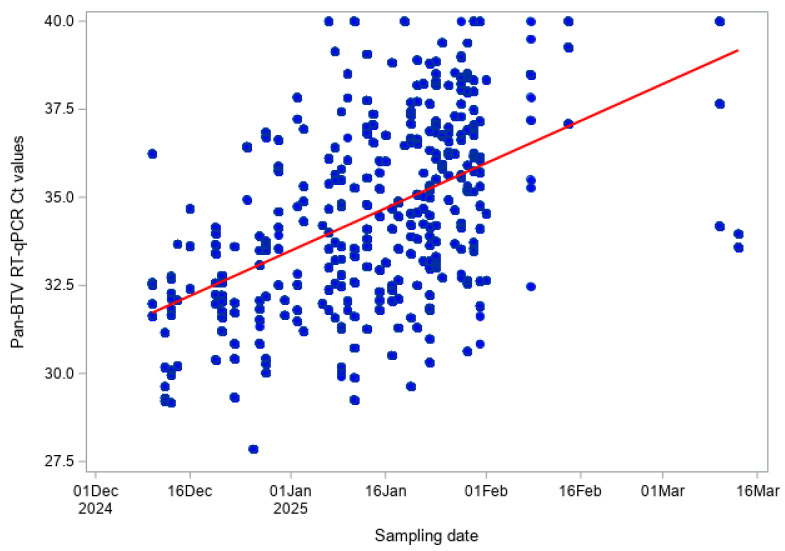
Pan-BTV RT-qPCR Ct values over time (BTV Hofmann RT-qPCR) in both cattle and sheep.

For the pan-BTV RT-qPCR, a moderate positive correlation between Ct values and sampling date was observed when considering both bovine and sheep samples combined (Pearson r = 0.520, *p* < 0.001; Spearman ρ = 0.536, *p* < 0.001; Figure 4). This indicates that Ct values tended to increase over the winter period, corresponding to a gradual decrease in viral RNA levels. The very low values (<0.001) indicate that the observed correlations are highly unlikely to have occurred by chance.

**Figure 5:**
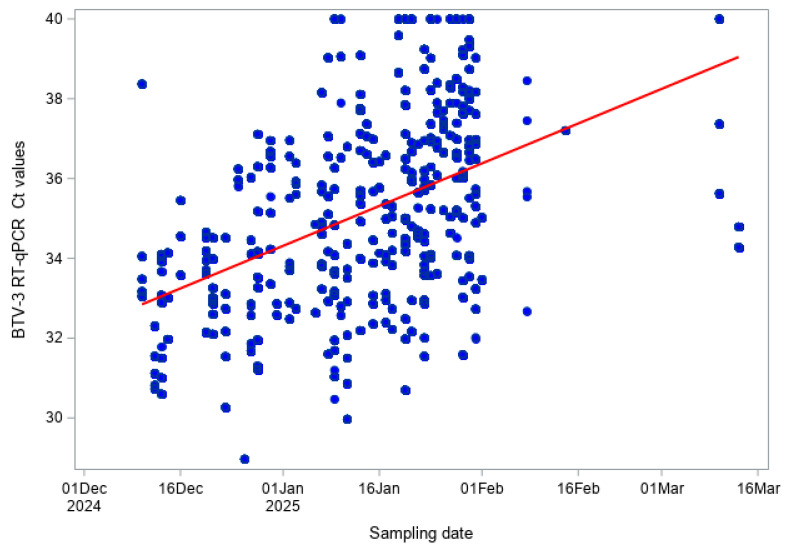
BTV-3 RT-qPCR Ct values over time in both cattle and sheep.

For BTV-3 RT-qPCR across both species, a similar pattern was observed (Pearson r = 0.473, *p* < 0.001; Spearman ρ = 0.504, *p* < 0.001; Figure 5), indicating a moderate and statistically significant increase in Ct values over time, consistent with decreasing viral RNA concentrations.

When species were analyzed separately, correlations were slightly stronger in cattle than in sheep.

**Figure 6:**
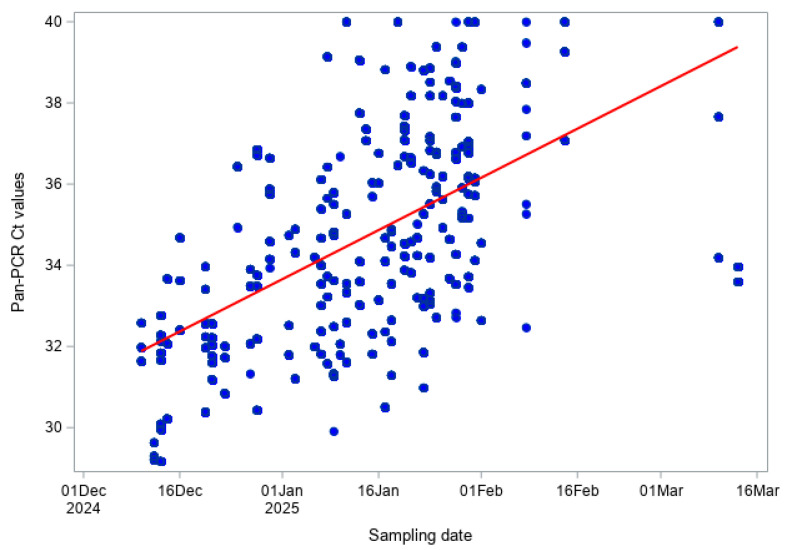
Pan-BTV RT-qPCR Ct values over time (BTV Hofmann RT-qPCR) in cattle.

**Figure 7:**
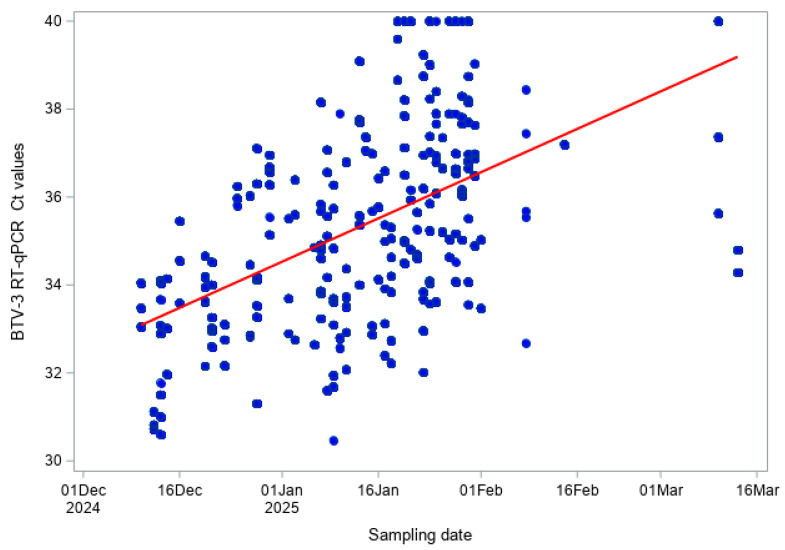
BTV-3 RT-qPCR Ct values over time in cattle.

**Figure 8:**
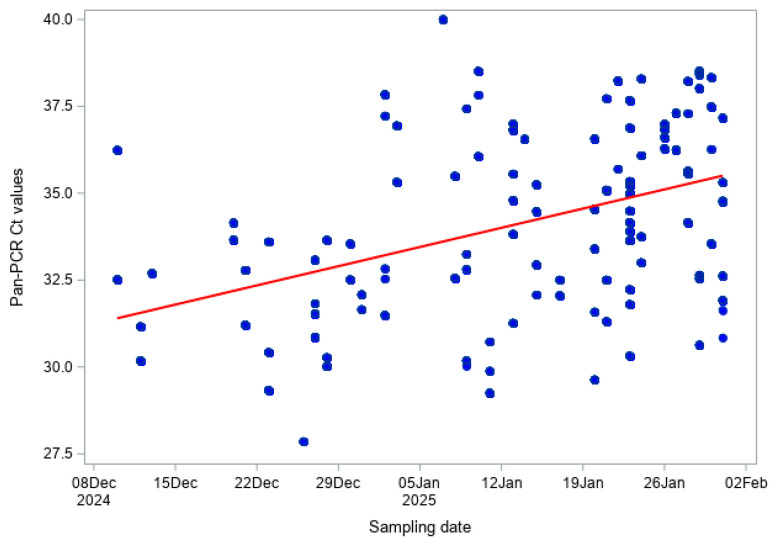
Pan-BTV RT-qPCR Ct values over time (BTV Hofmann RT-qPCR) in sheep.

**Figure 9:**
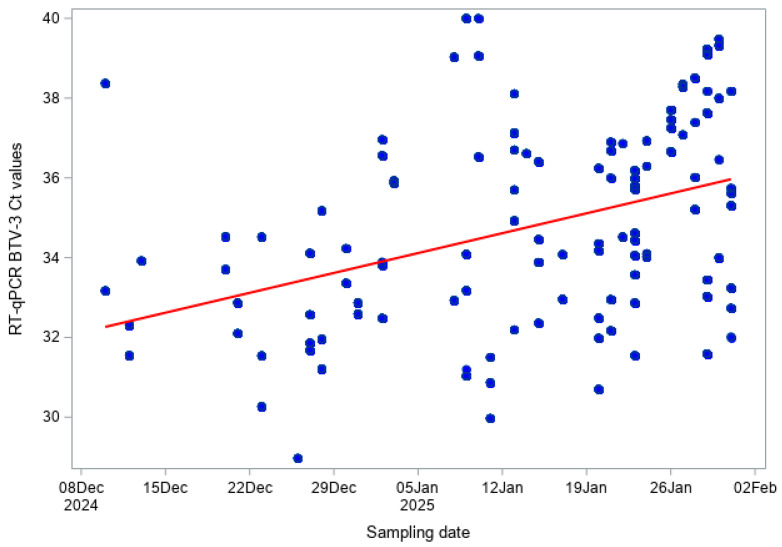
BTV-3 RT-qPCR Ct values over time in sheep.

In cattle, pan-BTV Ct values were moderately positively correlated with sampling date (Pearson r = 0.569, *p* < 0.001; Spearman ρ = 0.600, *p* < 0.001; Figure 6), and BTV-3 Ct values also showed a similar moderate positive trend (Pearson r = 0.518, *p* < 0.001; Spearman ρ = 0.562, *p* < 0.001; Figure 7). In sheep, correlations were slightly weaker but remained statistically significant: pan-BTV Ct values (Pearson r = 0.420, *p* < 0.001; Spearman ρ = 0.423, *p* < 0.001; Figure 8) and BTV-3 Ct values (Pearson r = 0.404, *p* < 0.001; Spearman ρ = 0.429, *p* < 0.001; Figure 9) showed a moderate increase over the study period.

## 4. Discussion

The results of the Belgian winter screening 2024–2025 clearly demonstrate that BTV-3 has rapidly and extensively disseminated across both cattle and sheep populations, as reflected by the very high herd seroprevalence observed in sampled herds (100% in cattle and 98.9% in sheep), highlighting an important level of viral transmission. For comparison, the herd-level prevalence in cattle was 3.4% in Belgium, as reported during the 2023–2024 surveillance campaign. Within-herd seroprevalence was ≥96% in cattle and ≥76% in sheep, indicating intense individual exposure. These figures reflect the high number of BTV-3 outbreaks officially reported in Belgium in 2024 [11]. The very high seroprevalence observed, despite the lower number of clinically reported cases, suggests that a substantial proportion of infections were likely subclinical. This highlights the importance of serological surveillance to accurately assess the true extent of virus circulation. Hainaut, West Flanders, and East Flanders exhibited the lowest apparent within-herd seroprevalence in both cattle and sheep. This difference is statistically significant in sheep between Hainaut and West Flanders, two provinces in western Belgium, compared to Liège, which is located in eastern Belgium. Based on tests sent for clinical suspicion of BTV cases (Sciensano Institute, NRL for bluetongue disease, available at https://moriskin.sciensano.be/shiny/bluetongue/, accessed on 26 January 2026), this spatial pattern may reflect a later exposure of herds in these western provinces during the epidemic wave, potentially resulting in a lower proportion of seroconverted animals at the time of sampling. Environmental and ecological factors, such as host density, vector habitat suitability, climatic conditions, and the presence of water bodies that favour Culicoides breeding, may also influence local transmission dynamics and could contribute to spatial heterogeneity in seroprevalence. However, given the near-universal herd-level exposure observed in this study, dedicated spatial and ecological analyses would be required to formally assess the role of these environmental determinants.

The use of ELISA with 100% sensitivity and specificity [26] provides high confidence in seroprevalence estimates. The ELISA used in this study detects antibodies directed against the VP7 protein and therefore reflects seroconversion following infection or vaccination, rather than directly measuring neutralizing antibodies. Neutralizing antibodies are primarily directed against VP2 and are responsible for serotype-specific protection. Consequently, ELISA results indicate prior exposure but do not directly measure protective immunity. Furthermore, the absence of BTV-8 and BTV-12 RNA in all RT-qPCR-tested samples supports indication of freedom from these serotypes during the 2024–2025 winter. This finding is particularly relevant given the reappearance of BTV-8 in previous years.

A minority of cattle herds (12/98; 12.2%) contained animals potentially vaccinated against BTV-3. Within these herds, seropositivity among vaccinated animals reached 83.2%, indicating substantial but incomplete herd immunity. This incomplete seroconversion might be due to the fact that some animals were sampled after a single vaccine dose, while a booster vaccination is required to achieve sufficient immunity as indicated in the product datasheets for Bultavo-3, Bluevac-3, and Syvazul BTV-3 in cattle. Also Van Leeuw [11] showed that a single dose BTV3 vaccination in sheep was insufficient to induce a strong neutralizing antibody response. In addition, the proportion of seropositive cattle after a single vaccine dose varied between vaccine products in our study, ranging from 74.8% for Bultavo-3 to approximately 90% for Bluevac-3 and Syvazul BTV-3. These differences should be interpreted cautiously, as the number of animals and herds vaccinated with each product was limited, and the interval between vaccination and sampling may have differed. Nevertheless, these findings further support that a single vaccine dose does not consistently induce detectable antibody levels in all animals. This highlights the importance of completing the full primary vaccination course, as recommended by manufacturers, to ensure adequate herd immunity and optimal protection against BTV-3. In the absence of a Differentiating Infected from Vaccinated Animals (DIVA) strategy, serological assays cannot distinguish vaccine-induced antibodies from antibodies induced by natural infection, which may further obscure within-herd comparisons. Some vaccinated herds may not have been officially recorded, potentially leading to an underestimation of within herd seroprevalence. Differences in vaccine formulations or field conditions at the time of BTV-3 vaccination could also have contributed to the observed lower seroprevalence compared with the national average. Previous experience with vaccination against another serotype (BTV-8) demonstrated a clear serological response, confirming the capacity of vaccination to induce detectable antibody production [10]. This pattern was also observed during the 2024 vaccination campaign against BTV-3 in the Netherlands [34,35].

Starting from 1 January 2025, in response to the BTV-3 outbreak, the Belgian Minister of Agriculture made vaccination mandatory for all sheep and cattle herds, requiring immunization against BTV-3 and BTV-8 (for cattle), with strict deadlines (e.g., 1 September 2025 for animals born before 2025) and financial support provided to farmers. The mandatory vaccination campaign rapidly achieved very high coverage nationwide, approaching 100% for BTV-3 and nearly 100% for BTV-8 [36]. Despite ongoing BTV-3 viral circulation in 2025, only one outbreak (BTV-8, 24 September 2025) was officially reported throughout the year 2025 [37], demonstrating that the BTV-8 vaccination campaign was effective in preventing clinical disease. However, the high BTV-3 seroprevalence suggests that herd immunity was already well established in non-vaccinated herds, indicating that mandatory vaccination was mostly useful for naïve animals, i.e., animals or herds which were not exposed to the virus during the 2024 epidemic and animals born in 2025.

Looking ahead, BTV-3 could re-emerge with renewed vector activity in spring 2026, if naïve animals are not protected by vaccination, highlighting the ongoing risk to both animal health and trade. In this context, sustained, coordinated surveillance across borders and seasons is essential. Furthermore, enhanced communication and engagement with farmers and veterinarians are needed to raise awareness and promote vaccination, particularly in sheep, which appear most affected clinically, but also in cattle to limit viral amplification as they are the preferred hosts for *Culicoides* spp. Continued vaccination in 2026 is therefore recommended, especially in naïve young animals born after the 2024 epidemic and mandatory 2025 vaccination campaign, to maintain herd immunity and mitigate the impact of BTV.

Finally, BTV-3 RT-PCR results indicate a consistent tendency for Ct values to rise over the winter, reflecting a reduction in detectable viral RNA in both cattle and sheep. The moderate positive Pearson and Spearman correlation coefficients confirm that this temporal trend is both linear and monotonic, and the highly significant *p*-values indicate that the observed associations are unlikely to have occurred by chance. This pattern is consistent with the expected decline of viral RNA during the vector-free winter period, when no new infections are expected. Slightly stronger correlations in cattle compared to sheep may reflect differences in susceptibility or sampling. The absence of detectable BTV-8 and BTV-12 RNA further supports the lack of active circulation for these serotypes. While Ct values provide a proxy for viral load rather than infectious virus, the large sample size and consistent correlations indicate a robust temporal trend. This temporal increase in Ct values has important implications for surveillance design and interpretation. As viral RNA levels decline over time, the sensitivity of RT-qPCR to detect previously infected animals is likely reduced later in the vector-free season. In addition, EDTA blood samples were tested in pools of ten to enable large-scale screening. Pooling may have reduced analytical sensitivity due to dilution and could therefore have slightly shortened the detection window for viral RNA, which should be considered when interpreting the RT-qPCR results. Therefore, winter screening programmes should ideally be conducted early in the vector-free period to maximize the probability of detecting residual viral RNA. Combining molecular and serological approaches remains essential to accurately assess virus circulation.

## 5. Conclusions

Belgium experienced the widespread exposure of its ruminant population to BTV-3 in 2024, as demonstrated by the very high herd and within-herd seroprevalence observed in cattle and sheep. During the same period, no active circulation of BTV-8 or BTV-12 was detected. These findings support the continued implementation of targeted surveillance and underline the importance of preparing for potential future BTV serotype incursions. It also shows that estimation of seroprevalence at the national level can be used as a tool for farmers, veterinarians, and animal health authorities to choose the best vaccination strategy.

## Figures and Tables

**Table 1:** Number of mixed herds to be sampled per Belgian province during the 2024–2025 bluetongue monitoring.

Region	Province	Rounded Number of 45 km × 45 km Grids Representing Each Province	Rounded Number of Herds to be Sampled in Each Province
Flanders	Antwerp	1.5	10
	East Flanders	1.5	10
	Limburg	1.0	7
	Flemish Brabant	1.0	7
	West Flanders	1.5	10
Wallonia	Hainaut	2.0	13
	Liège	2.0	13
	Luxembourg	2.0	13
	Namur	2.0	13
	Walloon Brabant	0.5	4
	TOTAL		100

**Table 2:** Distribution of cattle herds and cattle sampled per Belgian province during the 2024–2025 bluetongue monitoring.

Region	Province	Number of Herds Sampled	Number of Cattle Sampled and Included in the Analysis
Flanders	Antwerp	10	251
	East Flanders	10	279
	Limburg	7	172
	Flemish Brabant	7	186
	West Flanders	10	281
Wallonia	Hainaut	13	306
	Liège	13	324
	Luxembourg	12	346
	Namur	12	327
	Walloon Brabant	4	79
	TOTAL	98	2551

**Table 3:** Within-herd apparent seroprevalence in cattle at national, regional, and provincial level.

Level	Within-Herd Prevalence (%)	Lower 95% CI	Upper 95% CI
National level			
Belgium	94.6	91.8	96.5
Regions			
Flanders	95.5	91.8	97.6
Wallonia	93.8	89.1	96.5
Provinces			
Antwerp	97.1	90.7	99.2
East Flanders	91.8	81.6	96.5
Limburg	99.0	93.5	99.9
Flemish Brabant	99.5	97.0	99.9
West Flanders	91.9	78.1	97.3
Hainaut	89.6	75.6	96.0
Liège	92.2	83.9	96.4
Luxembourg	98.5	95.1	99.5
Namur	95.6	89.9	98.2
Walloon Brabant	99.4	95.8	99.9

**Table 4:** Distribution of sheep herds and sheep sampled per Belgian province during the 2024–2025 bluetongue monitoring.

Region	Province	Number of Herds Sampled	Number of Sheep Sampled and Included in the Analysis
Flanders	Antwerp	10	143
	East Flanders	10	170
	Limburg	7	133
	Flemish Brabant	6	79
	West Flanders	9	156
Wallonia	Hainaut	12	212
	Liège	10	190
	Luxembourg	10	180
	Namur	9	145
	Walloon Brabant	4	50
	TOTAL	87	1458

**Table 5:** Within-herd apparent seroprevalence in sheep at national, regional, and provincial level.

Level	Within-Herd Prevalence (%)	Lower 95% CI (%)	Upper 95% CI (%)
National level			
Belgium	85.5	80.4	89.5
Regions			
Flanders	84.0	75.6	89.9
Wallonia	87.3	81.5	91.5
Provinces			
Antwerp	84.0	52.9	96.1
East Flanders	81.6	66.4	90.9
Limburg	90.8	73.5	97.2
Flemish Brabant	88.0	61.1	97.2
West Flanders *	79.2	64.3	88.9
Hainaut *	76.6	59.6	87.9
Liège *	95.3	92.0	97.2
Luxembourg	84.7	74.2	91.4
Namur	94.5	87.4	97.7
Walloon Brabant	88.7	77.4	94.7

*: statistically significant differences were observed only between West Flanders and Liège, and between West Flanders and Hainaut (Tukey-adjusted *p*-values, see Table S2).

## Data Availability

Restrictions apply to the availability of these data. Data are available from the first authors with the permission of Belgian Federal Agency of the Food Chain and Sciensano.

## Supplementary Materials

The following supporting information can be downloaded at: https://www.mdpi.com/article/10.3390/v18030396/s1, Table S1. Pairwise comparisons of within-herd prevalence in cattle between provinces (Tukey-adjusted *p*-values). Table S2. Pairwise comparisons of within-herd prevalence in sheep between provinces (Tukey-adjusted *p*-values).

## Abbreviations

The following abbreviations are used in this manuscript:

ARSIA	Association Régionale de Santé et d’Identification Animales
BT	Bluetongue
BTV	Bluetongue virus
BTV-3	Bluetongue virus serotype 3
BTV-8	Bluetongue virus serotype 8
CI	Confidence interval
Ct	Cycle threshold
DGZ	Dierengezondheidszorg Vlaanderen
ELISA	Enzyme-Linked Immunosorbent Assay
GEE	Generalized Estimating Equation
ICC	Intra-cluster correlation coefficient
FASFC	Federal Agency for the Safety of the Food Chain
RT-qPCR	Quantitative real-time polymerase chain reaction
WS	Winter screening
WOAH	World Organisation of Animal Health
