# Supplementary material for: High Seroprevalence of Bluetongue Virus Serotype 3 in Belgian Cattle and Sheep After the 2024 Epidemic
Source: Viruses. 2026 Mar 22;18(3):396. doi: 10.3390/v18030396 (PMC13030625; doi:10.3390/v18030396)
Supplement: Supplementary file 1 [file viruses-18-00396-s001.zip › viruses-4166925-Supplementary Materials.pdf]

Supplementary Materials:

Table S1. Pairwise comparisons of within-herd prevalence in cattle between provinces (Tukey-adjusted p-values).

Province 1	Province 2	Adjusted P-value
Antwerp	East Flanders	0.92
Antwerp	Hainaut	0.81
Antwerp	Limburg	1.00
Antwerp	Liège	0.93
Antwerp	Luxembourg	1.00
Antwerp	Namur	1.00
Antwerp	Flemish Brabant	0.86
Antwerp	Walloon Brabant	0.94
Antwerp	West Flanders	0.96
East Flanders	Hainaut	1.00
East Flanders	Limburg	0.59
East Flanders	Liège	1.00
East Flanders	Luxembourg	0.40
East Flanders	Namur	0.99
East Flanders	Flemish Brabant	0.15
East Flanders	Walloon Brabant	0.31
East Flanders	West Flanders	1.00
Hainaut	Limburg	0.46
Hainaut	Liège	1.00
Hainaut	Luxembourg	0.27
Hainaut	Namur	0.94
Hainaut	Flemish Brabant	0.09
Hainaut	Walloon Brabant	0.22
Hainaut	West Flanders	1.00
Limburg	Liège	0.61
Limburg	Luxembourg	1.00
Limburg	Namur	0.93
Limburg	Flemish Brabant	1.00
Limburg	Walloon Brabant	1.00
Limburg	West Flanders	0.68
Liège	Luxembourg	0.40
Liège	Namur	0.99
Liège	Flemish Brabant	0.15

Liège	Walloon Brabant	0.32
Liège	West Flanders	1.00
Luxembourg	Namur	0.93
Luxembourg	Flemish Brabant	0.99
Luxembourg	Walloon Brabant	1.00
Luxembourg	West Flanders	0.57
Namur	Flemish Brabant	0.51
Namur	Walloon Brabant	0.71
Namur	West Flanders	1.00
Flemish Brabant	Walloon Brabant	1.00
Flemish Brabant	West Flanders	0.22
Walloon Brabant	West Flanders	0.39

Table S2. Pairwise comparisons of within-herd prevalence in sheep between provinces (Tukey-adjusted p-values).

**Province 1**	**Province 2**	**Adjusted P-value**
Antwerp	East Flanders	1.00
Antwerp	Hainaut	1.00
Antwerp	Limburg	1.00
Antwerp	Liège	0.85
Antwerp	Luxembourg	1.00
Antwerp	Namur	0.96
Antwerp	Flemish Brabant	1.00
Antwerp	Walloon Brabant	1.00
Antwerp	West Flanders	1.00
East Flanders	Hainaut	1.00
East Flanders	Limburg	0.99
East Flanders	Liège	0.08
East Flanders	Luxembourg	1.00
East Flanders	Namur	0.47
East Flanders	Flemish Brabant	1.00
East Flanders	Walloon Brabant	0.78
East Flanders	West Flanders	1.00
Hainaut	Limburg	0.91
Hainaut	Liège	0.01*
Hainaut	Luxembourg	0.99
Hainaut	Namur	0.18
Hainaut	Flemish Brabant	1.00
Hainaut	Walloon Brabant	0.90
Hainaut	West Flanders	1.00
Limburg	Liège	0.99
Limburg	Luxembourg	1.00
Limburg	Namur	1.00
Limburg	Flemish Brabant	1.00

Limburg	Walloon Brabant	1.00
Limburg	West Flanders	0.96
Liège	Luxembourg	0.09
Liège	Namur	1.00
Liège	Flemish Brabant	0.97
Liège	Walloon Brabant	0.70
Liège	West Flanders	0.02*
Luxembourg	Namur	0.61
Luxembourg	Flemish Brabant	1.00
Luxembourg	Walloon Brabant	1.00
Luxembourg	West Flanders	1.00
Namur	Flemish Brabant	1.00
Namur	Walloon Brabant	0.96
Namur	West Flanders	0.26
Flemish Brabant	Walloon Brabant	1.00
Flemish Brabant	West Flanders	1.00
Walloon Brabant	West Flanders	0.96

*: statistically significant (p-value <0.05)
